# Invasive Meningococcal Meningitis Serogroup C Outbreak in Northwest Nigeria, 2015 - Third Consecutive Outbreak of a New Strain

**DOI:** 10.1371/currents.outbreaks.06d10b6b4e690917d8b0a04268906143

**Published:** 2016-07-07

**Authors:** Jaime Chow, Kennedy Uadiale, Agatha Bestman, Charity Kamau, Dominique A. Caugant, Aminu Shehu, Jane Greig

**Affiliations:** Médecins sans Frontières, Sokoto, Nigeria; Nigeria Emergency Response Unit (NERU), Médecins sans Frontières, Sokoto, Nigeria; Médecins sans Frontières, Sokoto, Nigeria; Médecins sans Frontières, Amsterdam, Netherlands; WHO Collaborating Centre for Reference and Research on Meningococci, Norwegian Institute of Public Health, Oslo, Norway; State Primary Health Care Development Agency, State Ministry of Health, Sokoto, Nigeria; Manson Unit, Médecins Sans Frontières, London, United Kingdom

**Keywords:** disease outbreak, Epidemiology, meningococcal disease, Nigeria

## Abstract

BACKGROUND: In northwest Nigeria in 2013 and 2014, two sequential, localized outbreaks of meningitis were caused by a new strain of Neisseria meningitidis serogroup C (NmC). In 2015, an outbreak caused by the same novel NmC strain occurred over a wider geographical area, displaying different characteristics to the previous outbreaks. We describe cases treated by Médecins Sans Frontières (MSF) in the 2015 outbreak.

METHODS: From February 10 to June 8, 2015, data on cerebrospinal meningitis (CSM) cases and deaths were recorded on standardized line-lists from case management sites supported by MSF. Cerebrospinal fluid (CSF) samples from suspected cases at the beginning of the outbreak and throughout from suspected cases from new geographical areas were tested using rapid Pastorex® latex agglutination to determine causative serogroup. A subset of CSF samples was also inoculated into Trans-Isolate medium for testing by the WHO Collaborating Centre for Reference and Research on Meningococci, Oslo. Reactive vaccination campaigns with meningococcal ACWY polysaccharide vaccine targeted affected administrative wards.

RESULTS: A total of 6394 (65 confirmed and 6329 probable) cases of CSM including 321 deaths (case fatality rate: 5.0%) were recorded. The cumulative attack rate was 282 cases per 100,000 population in the wards affected. The outbreak lasted 17 weeks, affecting 1039 villages in 21 local government areas in three states (Kebbi, Sokoto, Niger). Pastorex® tests were NmC positive for 65 (58%) of 113 CSF samples. Of 31 Trans-Isolate medium samples, 26 (84%) tested positive for NmC (14 through culture and 12 through PCR); all had the same rare PorA type P1.21-15,16 as isolates from the 2013 and 2014 outbreaks. All 14 culture-positive samples yielded isolates of the same genotype (ST-10217 PorA type P1.21-15,16 and FetA type F1-7). More than 222,000 targeted individuals were vaccinated relatively early in the outbreak (administrative coverage estimates 98% and 89% in Kebbi and Sokoto, respectively).

CONCLUSIONS: The outbreak was the largest caused by NmC documented in Nigeria. Reactive vaccination in both states may have helped curtail the epidemic. A vaccination campaign against NmC with a long-lasting conjugate vaccine should be considered in the region.

## Background

Large-scale epidemics of invasive meningococcal meningitis in the African meningitis belt, a region of sub-Saharan Africa comprising 22 countries including Nigeria, have been attributed commonly to *Neisseria meningitidis* serogroup A (NmA), and less frequently to serogroups W and X.^1^^,^ 2 ^,^ 3 Epidemics in the meningitis belt occur in the dry season, which brings low humidity and dusty conditions and ends with the onset of rain.^4^^,^ 5 ^,^ 6 ^,^ 7 ^,^ 8 Since the introduction of the meningococcal A conjugate vaccine (*MenAfriVac*®), NmA cases have declined and NmA epidemics have been eliminated.^9^ However, in parallel, the proportion of cases and epidemics caused by other *Nm* serogroups such as W, X, and C has increased.

In 2013 and 2014, northwest Nigeria experienced two sequential outbreaks of meningitis in the adjacent Sokoto and Kebbi states caused by a new strain of *N. meningitidis* serogroup C (NmC).^10^ By contrast with NmA outbreaks, the NmC outbreaks were relatively localized and confined to small areas.^11^^,^ 12 In 2015, a meningitis outbreak occurred in the same region caused by the same novel NmC strain, but with different characteristics to the previous two outbreaks and wider geographic spread including a concurrent large outbreak in the neighbouring country of Niger where 8500 cases including 573 deaths were reported.^13^^,^ 14 We describe this unprecedented NmC outbreak in northwest Nigeria in the 2015 meningitis season.

## Methods

**Case Definition**

The following case definition for cerebrospinal meningitis (CSM) was used throughout the outbreak.^15^

- Suspected case of acute meningitis: sudden onset of fever with neck stiffness or petechial rash for adults and children over 1 year of age; sudden onset of fever with bulging fontanelle or petechial rash for children less than 1 year of age.
- Probable case of acute meningitis: a suspected case (as defined above) within an ongoing CSM outbreak or with cloudy cerebrospinal fluid (CSF) with or without a Gram stain.
- Confirmed case of acute meningitis: a suspected or probable case (as defined above) with positive CSF antigen detection via positive latex agglutination test or positive culture.

**Data Collection**

From February 10 to June 8, 2015, morbidity, mortality, and demographic data for CSM cases treated by MSF were recorded daily on a standardized line-list from each MSF supported case management site in Kebbi and Sokoto states. Only patients presenting for medical care at a case management site during this time period and who met the case definition were included on the line-list.

Within Kebbi and Sokoto states there are 21 and 23 local government areas (LGAs) respectively, and within each LGA there are approximately 10-15 wards, with variable population (estimated range 2,000-130,000). Population estimates for some affected wards in Kebbi state were provided by the Kebbi State Ministry of Health. All other population estimates were calculated using annual projections based on the most recent national census (2006). Weekly and overall incidence rates per 100,000 population were calculated using the combined population of all wards with cases from the start of the outbreak until that week.

The aggregated data analysed in this paper were collected as part of the routine activities that MSF has approval to conduct from the Ministries of Health. This work met the standards set by the independent MSF Ethics Review Board for retrospective analyses of routinely collected programmatic data.

**Laboratory Methods**

CSF samples were collected from suspected CSM cases at the beginning of the outbreak, as well as from suspected cases from new geographical areas during the outbreak. All samples were tested using the rapid Pastorex® (Bio-rad Laboratories USA) latex agglutination kit to determine the causative agent. Test kits were stored and transported at 2-8°C. Prior to usage, quality control tests on the kits were conducted.

A subset of CSF samples, being approximately 10 at the beginning of the outbreak and at least 3 from new areas during the course of the outbreak if practical, were also inoculated into Trans-Isolate medium^16^ within 1 hour of collection and sent to the WHO Collaborating Centre for Reference and Research on Meningococci, Oslo for confirmation testing. Briefly, Gram staining and standard biochemical reactions were done to identify bacteria, followed by serogrouping of *N. meningitidis* strains using Remel, GA, USA commercial antisera. Antimicrobial susceptibility was determined using minimal inhibitory concentrations (MIC) and classified using the European Committee on Antimicrobial Susceptibility Testing (EUCAST). Multilocus sequence typing (MLST) was done on each strain identified and compared to the MLST website. PCR analysis was done on culture-negative samples using QiAmp DNA mini kit (Qiagen) and analysed by real-time PCR for speciation followed by genogrouping. The PorA variant was determined by DNA sequencing of the *porA* gene using a nested *porA*-PCR.^17^ As part of routine practice, all cases who presented at an MSF-supported treatment site were tested for malaria infection using the rapid Paracheck-Pf® diagnostic test.

**Data Analysis**

Data from line-lists were entered weekly into an MSF standardized database in Microsoft Excel; quality checks and data validation were conducted weekly. Descriptive analysis including frequencies, summaries, and epidemic curves were produced using Microsoft Excel 2010.****

**Reactive Vaccination**

In Kebbi state, MSF supported two rounds of reactive vaccinations with meningococcal ACWY polysaccharide vaccine targeting individuals aged 2-30 years in wards that were affected at the time of making the vaccine request to the International Coordinating Group (ICG) on Vaccine Provision for Epidemic Meningitis Control. A subsequent round was conducted solely by the Kebbi State Ministry of Health targeting 1-29 year olds in wards newly affected as the outbreak spread. In Sokoto, MSF supported one round of reactive vaccination with meningococcal ACWY polysaccharide vaccine targeting individuals aged 2-30 years in wards affected at the time of making the vaccine request to the ICG. The second round was conducted solely by the Sokoto State Ministry of Health targeting 1-29 year olds in newly affected wards. In this paper, vaccination results are only reported from MSF-supported vaccinations.

## Results

**Case Counts and Rates**

Between February 10 and June 8, 2015, 6394 (65 confirmed and 6329 probable) cases of CSM were treated at MSF-supported treatment sites in Kebbi and Sokoto states (Table 1). The cumulative attack rate was 282 cases per 100,000 population in the affected wards. Treatment sites recorded 321 deaths, giving a case fatality rate (CFR) of 5.0%. Kebbi state treated the most patients, with 5714 cases (52 confirmed and 5662 probable) including 292 deaths (CFR: 5.0%), compared with Sokoto’s 680 cases (13 confirmed and 667 probable) including 29 deaths (CFR: 4.2%). However, the burden of illness was higher in the affected wards of Sokoto compared to Kebbi (attack rates: 317 vs. 279 cases per 100,000 population, respectively).

**Table 1. Number, deaths, and rates of confirmed and probable CSM cases treated by MSF in Kebbi and Sokoto States, February 10 – June 8, 2015:** *Population figures are based on the most recent census of the affected wards that had at least one case.

State	Population in Affected Wards*	Number of Confirmed Cases	Number of Probable Cases	Total Number of Cases	Cumulative Attack Rate (per 100,000 population)	Number of Deaths	Case Fatality Rate (%)
Kebbi	2,049,883	52	5662	5714	279	292	5.1
Sokoto	214,782	13	667	680	317	29	4.3
Total	2,264,665	65	6329	6394	282	321	5.0

**Case Demographics**

Of the 6394 cases treated, 3270 (51%) were female, 48% (3023/6354 [age missing for 40 cases]) were aged 5-14 years and 24% (1507) 15-29 years (Figure 1). Malaria co-infection was detected in 1358 (21%) cases. Of 4771 cases where immunization status for meningococcal meningitis C was recorded, 104 (2%) reported having received the meningococcal ACWY vaccine during the reactive vaccination campaign, of whom 88 (85%) presented their vaccination cards.

**Total number of cases and case fatality rate (CFR) by age group and sex of CSM cases treated by MSF in Kebbi and Sokoto states, February 10 – June 8, 2015:**
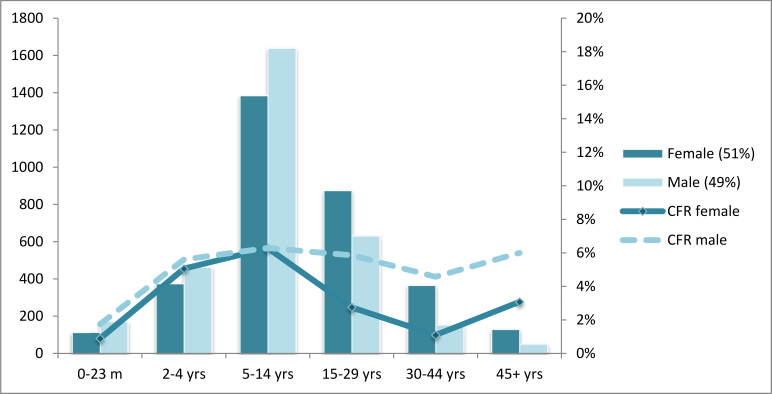
Total of 6354 cases with both age and sex recorded.

**Outbreak Description and Spread**

The outbreak started in epidemiological week 7 and lasted 17 weeks, with the highest number of cases (870; Figure 2) presenting for treatment by MSF in Kebbi state in week 18. This peak correlated with the opening of three more MSF-supported treatment sites in Kebbi. The epidemic threshold of 5 cases in one week in a localized area of population <30,000^15^ was first reached in a ward in Kebbi state in week 7 and a ward in Sokoto state in week 8. The incidence rate in the early-affected wards of Kebbi state peaked in week 9 and then remained relatively stable for the remainder of the outbreak. In Sokoto, the number of cases treated by MSF peaked in week 11 which also saw a large spike in incidence rate. However, this followed a rapid decline thereafter, and steadily decreased until the end of the outbreak. Both states saw a rapid decline in cases, particularly in Kebbi, from week 19, which closely followed the onset of rain in weeks 19 and 20.

**Number and weekly incidence rate of CSM cases treated by MSF in Kebbi and Sokoto states, February 10 – June 8, 2015:**
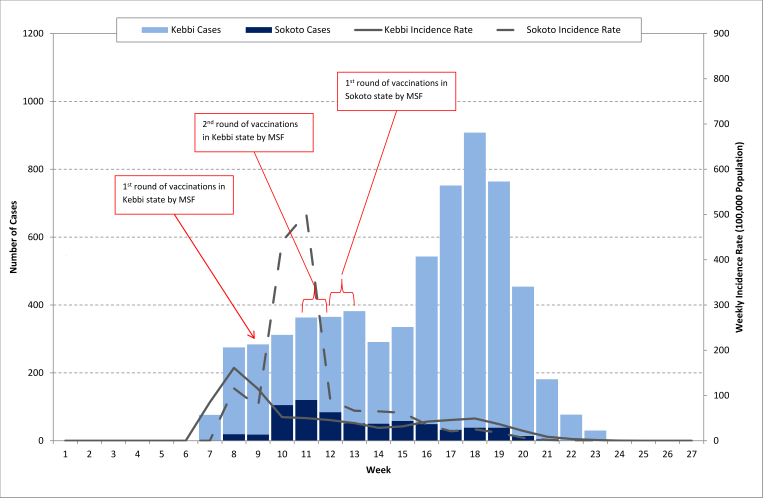
*Population estimates used to calculate incidence rates changed over the course of the outbreak as the population affected increased as the outbreak spread.

The outbreak spread over approximately 19,000km^2^, affecting 1039 villages within 113 wards in 21 LGAs in three states (Kebbi, Sokoto, and Niger) (Figure 3). The single case treated from Niger state was from a ward sharing a boundary with Kebbi.

**Cumulative attack rate by ward for CSM cases treated by MSF in Kebbi and Sokoto states, February 10 – June 8, 2015, Nigeria:**
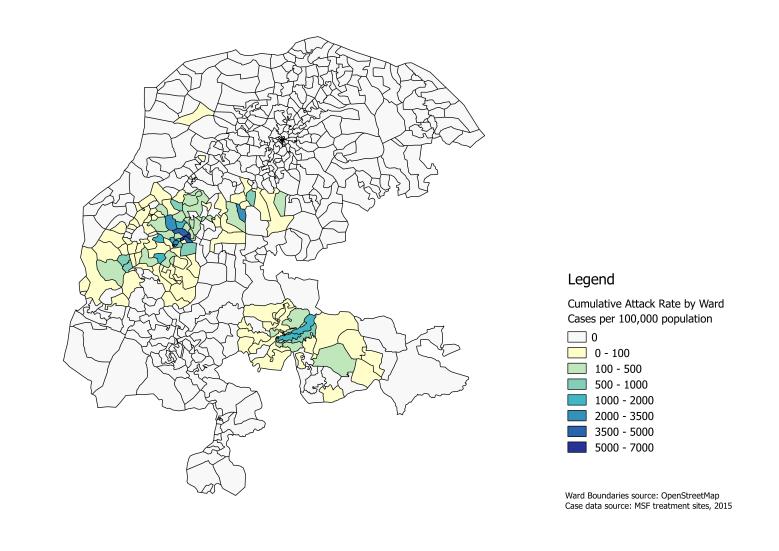

**Reactive Vaccination**

In Kebbi state, MSF-supported reactive vaccinations were held in weeks 9, 11, and 12 in 16 wards within four LGAs. More than 140,000 targeted individuals were vaccinated, providing an administrative coverage estimate of 98%. In Sokoto, MSF-supported vaccinations were conducted in weeks 12 and 13 in 10 wards within four LGAs. More than 82,000 targeted individuals were vaccinated, providing an administrative coverage estimate of 89%.

**Laboratory Results**

Of 113 CSF samples tested using Pastorex®, 65 (58%) were positive for NmC (52 [80%] from Kebbi; 13 [20%] Sokoto) and 48 (42%) were negative for any causative bacteria. Of 31 Trans-Isolate medium samples, 26 (84%) tested positive for NmC (14 through culture and 12 through PCR) and none tested positive for any other causative bacteria. All 26 NmC positive samples exhibited meningococcal DNA with the same genetic characteristics as isolates collected from the 2013 and 2014 outbreaks. The 14 culture-positive isolates were ST-10217 PorA type P1.21-15,16 and FetA type F1-7, while the 12 PCR-positive samples harbored the *porA* gene coding for type P1.21-15,16. By state, 14/18 (78%) samples from Kebbi and 12/13 (92%) from Sokoto were culture and/or PCR positive for NmC (Table 2).

**Table 2. Trans-Isolate test results conducted on CSF samples collected from patients treated by MSF in Kebbi and Sokoto states, by location, February 16 to March 26, 2015:** *All 26 samples had PorA type P1.21-15,16. The 14 culture-positive isolates had genetic sequencing of ST-10217 PorA type P1.21-15,16 and FetA type F1-7. **PCR stands for Real-time polymerase chain reaction.

	Kebbi State	Sokoto State	Total
All Trans-Isolate Tests (Culture and PCR**)			
Total number of CSF samples tested	18	13	31
Total number of positive NmC samples (%)	14 (78%)	12 (92%)	26* (84%)
Culture			
Number of CSF samples tested	8	6	14
Number of positive NmC samples (%)	8 (100%)	6 (100%)	14 (100%)
PCR			
Number of CSF samples tested	10	7	17
Number of positive NmC samples (%)	6 (60%)	6 (86%)	21 (71%)

## Discussion

We report on the largest outbreak of NmC ever recorded in Nigeria with 6394 confirmed and probable cases treated by MSF. This is almost 4000 cases more than the 2670 in Nigeria during 2015 included in the WHO meningitis bulletin, which also showed 8500 meningitis cases in neighbouring Republic of Niger.^18^ Our data show the continued expansion of NmC in the meningitis belt, increasing justification for a regional mass vaccination campaign with a long-lasting conjugate vaccine in order to help curtail further spread of NmC. The 2015 outbreak in northwest Nigeria was caused by the same NmC strain as the 2013 and 2014 outbreaks but caused more cases over a wider geographical area in Nigeria. NmC of the same strain (personal communication, DAC) was also identified from cases in the large concurrent meningitis outbreak in the Republic of Niger. The globally unique strain of NmC was first identified in Nigeria in 2013, and a WHO expert group meeting recently concluded that there is a high risk of continuing expansion of meningococcal meningitis in the meningitis belt, partly because of the emergence of this strain.^19^

Case numbers in 2015 were five times greater than the previous two outbreaks combined, and almost ten times more villages were affected. The spread of this outbreak was both rapid and geographically extensive, mimicking characteristics historically seen with NmA outbreaks.^11^^,^ 12 The 2015 outbreak followed typical meningitis seasonal patterns in the African meningitis belt, in that it began in the dry season following the Harmattan winds and stopped at the onset of rain.^4^^,^ 5 ^,^ 6 ^,^ 7 ^,^ 20 Age and sex distribution of cases were also consistent with those typically seen with meningitis outbreaks, with the 5-14 followed by 15-29 year age groups being the most affected and with equal proportions of males and females.^10^

The proportion (46%) of negative results for the Pastorex® latex agglutination tests was quite similar to previous outbreaks (64% [2013] and 48% [2014], respectively). This may be due to patients not volunteering a history of home management with antibiotics prior to presentation or showing signs of meningitis from non-bacterial causes. Similar to the 2013 and 2014 outbreaks, none of the CSF samples tested positive for NmA, which may show the effectiveness of the *MenAfriVac*® mass vaccination against NmA in this region in 2012 and 2013.

It is unclear why this outbreak was far greater in magnitude and spread than the two previous outbreaks. One explanation could be that it started in an urban area, facilitating transmission to more people than in the previous outbreaks, which were confined to small rural settings. Environmental factors also influence the pattern of meningitis,^4^^,^ 5 ^,^ 6 ^,^ 7 ^,^ 8 ^,^ 20 ^,^ 21 with epidemic cessation in Africa related to the onset of rain.^4^^,^ 5 ^,^ 6 ^,^ 7 In 2015 the onset of rain occurred later (week 19) than in previous years; in 2013 and 2014, the onset of rain occurred in weeks 18 and 15, respectively. Social-cultural factors are also believed to influence transmission patterns of meningitis; hypothetically, such behaviours could have differed in 2015 compared with the previous two outbreak years,^22^ but this was not assessed. Finally, vaccination uptake in Muslim Hausa communities may have been affected by vaccinee-vaccinator gender differences and the cultural requirement for male head-of-household consent to women being vaccinated. All these factors could have influenced rapid and widespread transmission of this outbreak.

The 2015 outbreak season marked the first reactive vaccination campaign against NmC in northwest Nigeria. In 2014, requests for vaccines against NmC were made by the Kebbi State Ministry of Health but were not approved by the ICG. In 2015, over 222,000 at-risk individuals were vaccinated in certain affected wards relatively early in the outbreak, which likely changed its course. However, a vaccine effectiveness study is needed in order to properly determine its impact. Due to the magnitude and rapid spread of the outbreak, reactive vaccination was not available for all affected wards, nor were there enough vaccines to reach herd immunity in some locations. As such, during future meningitis epidemic seasons it will be important that these areas are monitored closely, along with other locations that did not receive vaccination.

**Limitations**

The data used to describe and analyze the description of these cases and the outbreak was based solely on individuals who presented to an MSF-supported case management facility and were treated by MSF. Complete data related to NmC cases treated at non-MSF supported facilities were not available, and thus, were excluded from analysis. Furthermore, additional suspected NmC cases are likely to have been undetected and unreported. However, the proportion of cases not included in our report is believed to be low as MSF provided treatment to a large proportion of the affected areas that were reporting cases. Nevertheless, incomplete case ascertainment may mean that the characteristics of the cases and the outbreak described in this report are not perfectly representative of the entire cohort of NmC cases. However, the characteristics of the cases in this outbreak were consistent with those in the two previous NmC outbreaks in the region. It is possible that some suspected or probable cases were due to a different causative organism, as was the case in the concurrent outbreak in the country of Niger where NmW was also isolated,^14^^,^ 18 however NmC was the only organism identified in CSF samples tested using Pastorex®, PCR or culture.

**Conclusion and Recommendations**

This outbreak was the largest caused by *N. meningitidis* serogroup C ever documented in this part of the meningitis belt in northwest Nigeria. Since meningococcal meningitis ACWY polysaccharide vaccine should provide protection for at least 2 to 3 years,^23^^,^ 24 it can be anticipated that the geographic spread and case numbers should be reduced in the next few meningitis seasons. Nonetheless, to help further curtail outbreaks of NmC, a vaccination campaign with a long-lasting conjugate vaccine similar to *MenAfriVac*® should be considered in the region.

## Competing Interests

The authors have declared that no competing interests exist.

## Data Availability Statement

The underlying data is provided as a supplementary table in the manuscript.

## Supplementary Material

**Table S1. Number, deaths and weekly incidence rate of CSM cases treated by MSF in Kebbi and Sokoto states, February 10 – June 8, 2015:** 

		Kebbi			Sokoto			Total	
Epidemiological week	Cases (n)	Deaths (n)	Incidence	Cases (n)	Deaths (n)	Incidence	Cases (n)	Deaths (n)	Incidence
1	0	0	0	0	0	0	0	0	0
2	0	0	0	0	0	0	0	0	0
3	0	0	0	0	0	0	0	0	0
4	0	0	0	0	0	0	0	0	0
5	0	0	0	0	0	0	0	0	0
6	0	0	0	0	0	0	0	0	0
7	76	9	85	0	0	0	76	9	85
8	256	17	161	19	3	116	275	20	157
9	266	4	114	18	5	75	284	9	111
10	207	8	52	105	4	440	312	12	74
11	243	6	50	120	7	503	363	13	71
12	281	14	46	84	2	90	365	16	51
13	331	32	39	51	0	66	382	32	41
14	241	17	28	50	0	65	291	17	31
15	277	13	32	58	1	62	335	14	35
16	494	16	42	49	0	36	543	16	42
17	720	38	46	32	0	21	752	38	43
18	870	61	49	38	3	25	908	64	47
19	726	43	37	38	4	18	764	47	35
20	440	7	22	14	0	7	454	7	20
21	177	5	9	4	0	2	181	5	8
22	77	1	4	0	0	0	77	1	4
23	30	1	1	0	0	0	30	1	1
24	2	0	0	0	0	0	2	0	0
25	0	0	0	0	0	0	0	0	0
26	0	0	0	0	0	0	0	0	0
27	0	0	0	0	0	0	0	0	0
Total	5714	292	279	680	29	317	6394	321	282

**Table S2. Number, deaths and rates of CSM cases treated by MSF, by patient residence, February 10 – June 8, 2015**

Supplementary-table-2.pdf
